# A Qualitative Study of HR/OHS Stress Interventions in Australian Universities

**DOI:** 10.3390/ijerph15010103

**Published:** 2018-01-09

**Authors:** Silvia Pignata, Anthony H. Winefield, Carolyn M. Boyd, Chris Provis

**Affiliations:** 1 Asia Pacific Centre for Work, Health and Safety, University of South Australia, Adelaide, SA 5000, Australia; anthony.winefield@unisa.edu.au; 2 School of Engineering, University of South Australia, Adelaide, SA 5000, Australia; cmb@internode.on.net; 3 School of Psychology, University of Adelaide, Adelaide, SA 5000, Australia; 4 School of Management, University of South Australia, Adelaide, SA 5000, Australia; chris.provis@gmail.com

**Keywords:** work stress, stress interventions, universities, well-being, leadership

## Abstract

To enhance the understanding of psychosocial factors and extend research on work stress interventions, we investigated the key human resource (HR)/occupational health and safety (OHS) stress interventions implemented at five Australian universities over a three-year period. Five senior HR Directors completed an online survey to identify the intervention strategies taken at their university in order to reduce stress and enhance employee well-being and morale. We also explored the types of individual-, organization-, and individual/organization-directed interventions that were implemented, and the strategies that were prioritized at each university. Across universities, the dominant interventions were strategies that aimed to balance the social exchange in the work contract between employee-organization with an emphasis on initiatives to: enhance training, career development and promotional opportunities; improve remuneration and recognition practices; and to enhance the fairness of organizational policies and procedures. Strategies to improve work-life balance were also prominent. The interventions implemented were predominantly proactive (primary) strategies focused at the organizational level and aimed at eliminating or reducing or altering work stressors. The findings contribute to the improved management of people at work by identifying university-specific HR/OHS initiatives, specifically leadership development and management skills programs which were identified as priorities at three universities.

## 1. Introduction

At an international level, “strong university sectors are associated with stronger economies and higher standards of living” as they contribute markedly to national economic and social prosperity [1] (p. vi). As Australia’s university sector delivers approximately $25 billion to the economy per annum [1], it is crucial that the university sector and its workforce thrive in order to continue to deliver high quality research and teaching outcomes to students. Yet, work stress has increased in the sector, hand in hand with increased competition among universities, technological changes and casualization of the workforce. Job-related stress, defined as “the adverse reaction people have to excessive pressure or other types of demand placed on them” [2] (p. 7), is one of the largest issues in workplaces globally [3]. Due to its adverse impact on worker health and organizational performance [4], it is important to investigate the management of work stress.

A stress management intervention has been defined as “any activity, program, or opportunity initiated by an organization, which focuses on reducing the presence of work-related stressors or on assisting individuals to minimize the negative outcomes of exposure to these stressors” [5] (p. 252). Despite the wide variety of programs to manage stress and improve employee well-being and morale, little evaluative research on organizational stress interventions has been reported in the literature [6]. Reasons for this relate primarily to the methodological and conceptual challenges inherent in stress-intervention research [7,8], the high cost and complexity of organizational interventions [9], and the mixed results of reviews and research on the effectiveness of organizational health interventions [10]. Indeed, Karanika-Murray, Biron, and Saksvik [10] (p. 255) assert that “there is still a great divide between what organizations do to promote workplace health and well-being, on one hand, and what researchers know in terms of what causes ill-health at work, on the other”. Similarly, Cox, Taris and Nielsen [11] call for researchers to examine innovative approaches to improving employee health and well-being. In order to provide solutions to the causes of work-related stress, there is a need for applied researchers to investigate what types of interventions work, when are they effective, and why and how organizational level stress interventions work [12]. We address the underlying question of what university management thinks will work (i.e., their implicit theories of the causes of stress and what will be effective in reducing stress) by examining the types of organizational stress initiatives that were implemented in the specific context of Australian universities, an area experiencing high levels of work stress.

Empirical research suggests that for stress interventions to be effective: (1) the development of interventions should include a participatory approach and involve, and be supported by, employees, unions, and senior management [13]; (2) interventions should publicly detail the process involved in their implementation as doing so can play a salient role in their effectiveness; (3) the intervention strategies should include multilevel approaches that combine individual- and organizational-directed stress management and reduction strategies [14]; (4) interventions should have clearly defined assessment/monitoring policies; and (5) the evaluations of interventions should use multiple self-report and objective measures [12]. However, there are difficulties in evaluating organizational interventions, as the few published successful intervention studies are often methodologically unsound [15]. The goal of this study therefore is to understand the implicit theories of stress management demonstrated by university management. Using a qualitative method, the study identifies the processes and measures to reduce or manage stress undertaken by Human Resource (HR) Directors at five Australian universities across two time-points.

Work stress interventions are delineated into the classifications of primary, secondary, or tertiary approaches that may target the individual, the organization, or both the individual, and the organization. LaMontagne, Keegel, Louie, Ostry, and Landsbergis [16] assert that comprehensive interventions that encompass primary, secondary, and tertiary approaches are more effective on individual and organizational outcomes than other programs, as they address the causes and the consequences of stress by targeting the organization’s and individuals’ needs. Person-directed (individual) interventions, such as cognitive-behavioral therapy and employee assistance programs (EAPs) [17], focus on the employee, or group, to improve their coping resources; the aim is to assist them to deal more effectively with demanding situations, or to modify their appraisal of specific work-related stressors to reduce the perception of threat and its associated aversive emotional responses [18]. Other strategies include organizational (work-directed) interventions that focus on the work environment, and are often referred to as stressor reduction processes, and include job redesign, selection, placement, training, and education programs [17]. 

Interventions directed at the individual-organization interface focus on the interplay between individual employees and the organization; they include management initiatives such as co-worker support groups to improve relationships at work, improving person-environment fit, addressing role issues, and increasing workers’ participation and autonomy [19]. Giga, Noblet et al. [17] assert that a combination of multilevel organizational and individual intervention strategies is most effective in reducing stress as such an approach addresses the organizational environment, the individual and the individual-organization interface. This view is supported by reviews by Richardson and Rothstein [20], and Tetrick and Winslow [21].

To determine the level at which the HR intervention strategies investigated in the present study were targeted, we categorized them as individual (person-directed), organizational (work-directed), and individual-organization interface-directed, in accordance with the DeFrank and Cooper [22] classification of occupational stress management programs. This classification approach assists in the evaluation of the intervention strategies and their application in university workplaces. To understand the nature of the work-stress process, and gain an insight into the relation between psychosocial risk factors (any organizational factors and interpersonal relationships in the work setting that may affect the health of workers), occupational stress and stress-reduction strategies, it is necessary to examine them within the framework of work-stress models. As Schaufeli and Taris [23] (p. 59) assert that the job demands-resources (JD-R) model “is perfectly suited to guide the integration of occupational health and human resources policies in organizations”, the conceptual framework of the JD-R model [24] will be employed in the present study.

The tenet of the JD-R model is that high job demands lead to strain and health impairments, and that abundant job resources are instrumental in achieving work goals as they lead to increased motivation and higher productivity [23]. The model is an open heuristic model that provides a parsimonious description of the way that two key factors (demands, resources) within the work environment can contribute to job strain or conversely, to improving employee morale and engagement [25]. Job demands are the physical, psychological, social, or organizational aspects of the job (i.e., workload, time pressure, role conflict) that require sustained physical, and/or cognitive and emotional effort associated with physiological and/or psychological costs [24]. Alternatively, job resources are the physical, psychological, social, or organizational aspects of the job (i.e., feedback, recognition, job control, social support) that may: reduce job demands and the associated physiological and psychological costs; aid in achieving work goals; meet intrinsic needs for relatedness; or stimulate personal growth, work engagement, learning and development (the motivation process) [26]. Hence, increased opportunities for personal growth, learning, and development (the motivational hypothesis) link job resources to beneficial individual and/or organizational outcomes through improved morale and positive work attitudes (job satisfaction, work engagement, organizational commitment). For example, Dollard and Bakker [27] present longitudinal evidence that an organization’s policies, practices and procedures to protect workers’ psychosocial health and safety (termed Psychosocial Safety Climate: PSC) are an organizational resource that influences the work context. As job resources are negatively related to stress [24] and are assumed to reduce job demands, work environments that offer many resources can reduce stress and foster morale. Thus, we argue that the stress interventions implemented by university management are an organizational resource component within the JD-R model. The new PSC theory [27] taps the issue of management priority for worker psychological health versus productivity imperatives. The theory proposes that PSC is influenced by senior management and their values, and is a precursor to working conditions. 

Research on university staff has shown that work-related stress in universities has risen internationally due to the external pressures facing universities and their workforce including increased competitiveness, the implementation of policies to measure research performance, broader technological advances, demand-driven funding [28] and reduced government financial support [29,30,31,32,33,34,35]. Specific to the Australian context, the adverse impact of economic and management imperatives on the well-being of staff in Australian universities has been well documented [36,37].

The present research emerged from a qualitative focus group study of 15 universities which showed that both academic and non-academic staff experienced high levels of stress associated with: “(1) a lack of funding, resources and support services; (2) task overload; (3) poor leadership and management; (4) a lack of promotion, recognition and reward; and (5) job insecurity” [37] (p. 68). Consequently, a two-wave longitudinal study of occupational stress was undertaken in which the first phase involved surveying all contract and tenured staff at 17 universities (the responding sample of 8732 participants provided a 25% response rate). The study found that reduced staffing levels and job security, and increased student numbers were related to high levels of strain and low levels of perceived control in staff [36]. Based on those findings and empirical research on work stress, the researchers made recommendations to enable the participating universities to introduce multilevel stress-reduction interventions that included raising staff awareness of EAPs, increasing the fairness of university procedures (promotion, redundancy, performance appraisal), enhancing communication and consultation processes, reducing academics’ job demands, and increasing job security [36]. Three years later, a follow-up survey was undertaken at 13 universities with responses from 6321 participants (a 26% response rate).

The present study examines information supplied by HR Directors at five of those 13 universities. The assumption motivating this qualitative examination is that a more specific and in-depth examination of the five individual universities will reveal details of the specific and/or customized intervention strategies that were implemented in the period between the initial and the follow-up study, and how the interventions were targeted. Investigating these characteristics may provide an insight into how university management perceives psychosocial risk factors in university employees, and how management attempts to address those risk factors. The present study contributes to the applied stress intervention literature by examining the following research questions from the perspective of university management: (a) the types of HR/OHS interventions and strategies that were implemented by the universities that were designed to reduce employee-levels of work stress and increase morale and well-being; (b) the types of individual-, organization-, and individual/organization interface-directed interventions that were implemented; and (c) the key intervention strategy that was implemented at each university that best exemplified the university’s commitment to improving staff well-being and morale. Accordingly, we investigated the following research questions using a qualitative method in real-life university settings.

Research Question 1 (RQ1): What intervention strategies were employed by university management?
Research Question 2 (RQ2): At what level were the interventions directed?
Research Question 3 (RQ3): What initiatives were implemented as a priority?

## 2. Materials and Methods

In 2007, the Vice-Chancellors of the 13 universities who participated in the follow-up survey were contacted and invited to participate in the present study. Five Vice-Chancellors agreed to participate whilst eight Vice-Chancellors declined the invitation. The five participating universities located in three Australian States comprised: two “Old” universities, two “New” universities, and one “Australian Technology Network (ATN)” university. The eight non-participating universities constituted one “Old”, two “New”, two “ATN” and three “Middle” universities. In Australia, “Old” universities are those established between 1853 and 1911, and “Middle” universities refer to those established between 1954 and 1974. “New” universities are those established between 1988 and 1992 [36], and “ATN” universities are former Institutes of Technology that are now focused on industry collaboration and applied research [38].

Each Vice-Chancellor nominated a Senior HR Director to participate in the research. The five nominated Directors were contacted via email and each agreed to participate in the study and complete an Intervention Evaluation Survey (IES) in 2008. Ethical approval (protocol P019/08) was granted on 4 April 2008 by the University of South Australia’s Human Research Ethics Committee.

The IES was based on empirical evidence and research by Pignata and Winefield [39], that identified the specific HR/OHS stress management and reduction interventions that were undertaken at one Australian university. Using a participative/collaborative approach, a preliminary draft of the IES was circulated to all of the nominated university representatives in order to obtain their feedback and to develop the survey further. After including their feedback, the survey was then examined by an independent university HR Director who provided feedback on the survey content only, and did not participate in the study. This feedback was also incorporated into the IES.

The IES comprised two sections. Section 1 consisted of a checklist of 11 intervention targets and listed multiple intervention options. The intervention targets and examples of their respective options included: (1) increase awareness of stress and its management—eight options (e.g., promoted employee assistance program to staff); (2) enhance job design and reduce job pressure—13 options (e.g., streamlined administrative procedures); (3) improve work-life balance in staff—10 options (e.g., established/promoted family-friendly provisions); (4) improve communication and consultation with staff—eight options (e.g., improved all communication processes from management to staff); (5) address tenure concerns—four options (e.g., established/reviewed vocational counselling for redeployees); (6) enhance workplace interpersonal relations—six options (e.g., implemented/reviewed workplace bullying and violence policy); (7) enhance the fairness of policies and procedures—11 options (e.g., increased transparency of performance appraisal procedures); (8) increase staff levels of trust in senior management—eight options (e.g., developed leadership capabilities in staff); (9) enhance training, career development and promotional opportunities—15 options (e.g., established mentoring/coaching program); (10) improve remuneration and recognition practices—13 options (e.g., implemented/reviewed performance planning and review systems); and (11) improve health and lifestyle in staff—seven options (e.g., established/reviewed fitness and exercise program).

Section 2 of the IES was an open-ended questionnaire in which senior HR Directors were asked to describe a key strategy (program, policy or procedure) that best exemplified their university’s commitment to improving staff well-being and morale. They were also asked to provide details of the strategy’s aim, the extent of its coverage, the target group(s), how the at-risk group(s) were identified, and which groups were involved in planning and implementing the strategy.

To investigate the research questions, data analyses were conducted independently for each university, both to relate back to the objectives and to draw out policy implications. A detailed investigation of each case allowed us to draw out any trends and provided the basis for cross-case comparison [40]. Microsoft Excel was used to chart the data. Following the techniques proposed by Yin [41], once each case was completed, the results were checked to see if they replicated the findings in the previous cases. Once all the cases were entered into the data set, cross-case conclusions were then drawn.

## 3. Results

Table 1 lists the key types of HR/OHS intervention strategies undertaken at the five universities within a three-year period. In order to determine the level at which the interventions were targeted, the intervention strategies were classified into the categories of individual, organization, or individual/organization level interventions.

As shown in Table 1, the key issues targeted by HR management across the universities involved strategies to enhance training, career development and promotional opportunities for staff (35 strategies); and to improve remuneration and recognition practices (35 strategies). Other salient strategies aimed to improve work-life balance (29 strategies), and to enhance the fairness of policies and procedures (27 strategies). The total number of HR/OHS interventions implemented at each university during this period ranged from 37–51 strategies. It is of interest that the highest percentages of strategies implemented at Universities D and E were those targeting training, career, and promotion areas (19.6%) and (18.8%), respectively. In contrast, Universities B and C focused on enhancing employee remuneration and recognition, (20.5%) and (29.7%), respectively. The highest percentage of strategies at University A were those aimed at enhancing employees’ work-life balance (19.5%).

Table 1 also shows that the majority of interventions implemented were focused at the organizational level and aimed at eliminating, reducing or altering stressors at work. These strategies included: improving training, career, and promotion opportunities; enhancing remuneration and recognition practices, and work-life balance strategies; enhancing procedural justice, and increasing trust in senior management; redesigning jobs; enhancing communication and consultation processes; and addressing job security and tenure concerns. In terms of the JD-R model, strategies such as improving training, career, and promotion opportunities were aimed at building resources, whilst interventions directed at job redesign and job pressure were stress management strategies to reduce job demands. Strategies to increase employee awareness of stress and the management of stress were classified as both individual- and organization-directed interventions, and strategies to enhance interpersonal relationships were the only individual-organization interface-directed strategies that were implemented. In addition, interventions addressing employee health and lifestyle factors were the single type of individual-directed strategies that were implemented.

The HR Director at each university also provided details of the specific (priority) strategy implemented at their university that best exemplified the university’s commitment to improving staff well-being. Moreover, they listed each strategy’s aim, extent of coverage, target group, and the planning and implementation process that was undertaken in order to appreciate fully the extent of the intervention strategy.

Table 2 shows that the majority of priority strategies implemented at three universities involved introducing leadership development and management skills programs to enhance the capacity of leaders, specifically Heads of Departments, to manage their employees. The principal aims of these strategies were to improve the ability of supervisors and managers to deal effectively with staff to improve staff commitment and morale, and to improve outcomes, including the management of work stress. The priority intervention implemented at a fourth university was a university-wide strategy enabling early intervention in and management of stress claims, in order to increase awareness of the resources available to staff (and supervisors) and to assist in the early identification and reporting of stress and stressful situations. The fifth key priority intervention strategy was a university-wide restructuring of academic portfolios to streamline systems and administration processes within the university.

Each HR Director provided details of the specific strategies designed and undertaken at their university to improve employee well-being and morale within the categories of: (1) raising the awareness of stress; (2) re-designing jobs or reducing work pressure; (3) addressing tenure concerns; (4) improving work-life balance; (5) improving communication and consultation processes; (6) enhancing interpersonal relationships; (7) increasing levels of trust in senior management; (8) enhancing training and promotional opportunities; (9) enhancing fair procedures; (10) improving remuneration and recognition practices; and (11) improving employee health and lifestyle (see Table 3, Table 4, Table 5, Table 6 and Table 7).

In terms of strategies to raise awareness of stress and its management, Table 3 shows that each university updated and promoted EAPs or Critical Incident Stress Management (CISM) programs to assist employees to deal with the normal physical and emotional reactions that may result from critical incidents at work. Job redesign strategies to streamline administrative processes and to assist new employees featured strongly at four of the universities (it should be noted that University C was not able to provide a response regarding job redesign strategies). Improved communication processes regarding the renewal of staff contracts were undertaken by the majority of the universities.

Table 4 shows that interventions to improve work-life balance were widespread, as all universities established or promoted family-friendly policies and working arrangements, and four universities encouraged staff to take accrued leave. Strategies to improve consultation and enhance communication processes from management to staff were implemented at the majority of the universities.

Table 5 lists the strategies implemented to enhance interpersonal relationships and increase staff levels of trust in senior management. The updating of equal opportunity, workplace bullying and violence, and discrimination and harassment policies were undertaken by the majority of the universities. A focus on leadership development and mentoring of staff for leadership positions was highlighted by four of the universities.

As can be observed in Table 6, interventions to enhance training, career development and promotional opportunities for staff, particularly management, were the predominant strategies undertaken by all of the universities. Specifically, management-directed training in performance management, change management, time management, interpersonal skills, and training for middle managers in management skills were widespread across all of the universities.

Table 7 shows that the majority of the strategies to enhance the fairness and openness of procedures were those to review recruitment, selection and appointment policies; review promotion procedures for academic staff; provide clear promotion criteria; and update the performance management system. All five universities implemented multiple strategies to recognize staff achievements and improve remuneration practices. Chief among these were updating performance planning and review systems, developing or improving processes for recognizing excellence in teaching, and acknowledging staff in teaching awards or grants. Only two universities attempted to enhance employees’ health and lifestyle directly by implementing meditation or fitness and exercise programs.

## 4. Discussion

The key purpose of this study was to address the gap in existing applied intervention research by investigating the stress interventions that were implemented by management at five universities between two time-points in order to gather details of the types of strategies (RQ1), how they were targeted (RQ2), and which interventions were regarded as priority strategies (RQ3). Firstly, across the universities, the dominant interventions were strategies that aimed to balance the social exchange in the work contract between employee-organization as highlighted in the effort-reward imbalance model [42]. For example, there was an emphasis on initiatives to: enhance training, career development and promotional opportunities for staff; improve remuneration and recognition practices; and to enhance the fairness of organizational policies and procedures. Strategies to improve work-life balance for staff were also prominent. Given previous research and the recommendations made to the universities by Winefield and colleagues [36,37], we had expected that university management would target strategies to those areas. 

The total number of HR/OHS interventions implemented at each university during this period ranged from 37–51 strategies (see Table 1). The largest number of interventions were implemented by Universities D and E, which adopted stronger top-down strategies than the other three universities, with a third of the strategies for both universities targeting the psychosocial work environment aspects of job design; and training, career, and promotion opportunities. As organizational-level strategies “can be truly preventative, tackling workplace and organizational issues at their source” [10] (p. 255), it is important that management target those areas. With regard to the JD-R framework, organizational climate or PSC are also top-down influences, as PSC is positively linked to employee engagement and psychological well-being [43] via job design (the levels of demands and resources). As jobs are designed in harmony with management strategy and choice [44], in psychologically healthy workplaces (i.e., a high PSC workplace) we expect that jobs are designed where demands are manageable, and resources are adequate. 

Given that a comprehensive review of work stress preventive interventions by Kompier and Kristensen [45] showed that the majority of interventions to prevent mental health problems at work are individual and reactive strategies, it is noteworthy that the intervention strategies implemented by university management were predominantly primary intervention strategies focused at the organizational level and aimed at eliminating, reducing or altering stressors at work. The strategies included: improving training, career, and promotion opportunities; enhancing remuneration and recognition practices and work-life balance strategies; enhancing procedural justice, and increasing trust in senior management; redesigning jobs; and enhancing communication and consultation processes. It should be noted that University C did not provide details of any interventions related to job design, tenure, communication and consultation, or trust in senior management. 

In response to the second research question, each of the participating universities implemented multilevel intervention strategies to improve employee well-being and morale, which is consistent with prior research that shows that the most effective intervention strategies are multilevel interventions that combine organizational (work-directed) as well as individual (person-directed) strategies [13,16,45]. Thus, in order to understand work stress and employee engagement, a multilevel approach to occupational health needs to take both management and individuals’ needs into consideration. 

With regard to the third research question, the results of the present study show that the key issues targeted by HR management across the universities were proactive organization-directed strategies to enhance the capacity of leaders, specifically Heads of Departments, to manage employees, improve employee commitment and morale, and to improve outcomes including the management of stress (see Table 2). Such strategies were listed as priorities at three of the five universities (A,D,E). In addition, senior management at University C implemented a university-wide strategy for the early intervention and management of stress claims in order to increase an awareness of the resources available to staff (and supervisors) and to assist in the early identification and reporting of stress and stress situations. 

In terms of the JD-R theoretical model, increasing job resources in the form of enhancing employees’ training and career development opportunities, and improving remuneration and recognition practices may lead to increased opportunities for personal growth, learning and development (the motivational hypothesis), and to positive organizational outcomes through increased morale and positive work attitudes (i.e., increased commitment to the organization). It is of interest that a recent study of more than 15,000 employees from over 1200 workplaces [46] found that targeted HR training practices indirectly influenced organizational productivity through employee perceptions of job resources and job satisfaction. 

It is intuitively appealing to consider that implementing interventions should reduce work stress. However, it is necessary to examine the discrete context in which employees are working, and to acknowledge process issues in organizational stress and well-being interventions [6]. Indeed, on the basis of eleven European case studies, Kompier and Cooper [47] emphasize that interventions need to be context-specific and accurately assess both individual and organizational factors which are crucial to the intervention’s success. Some intervention strategies may have led to desirable employee and organizational outcomes, which has practical implications for university management, particularly HR/OHS management, as the motivating potential of job resources should be acknowledged and exploited. Beneficial outcomes for both the employee and the organization may be achieved if organizations implement targeted strategies to increase well-being and morale, and communicate and promote the measures taken by HR/OHS departments to employees. 

## 5. Conclusions

It appears that university managements’ resources were targeted specifically to increase employee perceptions of procedural justice and leadership development and training. The interventions implemented were predominantly proactive (primary) strategies focused at the organizational level. The results imply that a focus on stress prevention and management and improving the quality of the relationships between employees and senior management in terms of increased strategies to promote procedural justice, trust and commitment to the organization, may lead to positive HR benefits for employees and organizations. 

However, there are limitations that need to be acknowledged. First, our intervention study obtained objective evidence from university management of university-wide interventions, but there may have been variations between departments in the type or extent of the stress-reduction strategies undertaken. Hence, further research should be aimed at a departmental level using an appropriate sample size to investigate the specific faculties/organizational units within each university, and obtain details of their targeted interventions. 

In the university context, work-related stress has been linked to economic pressures, internationalization, the growing use of technology, and demand-driven funding [28]. As these factors may also impact other industries that employ knowledge workers who develop and apply specific knowledge (e.g., scientists, educators, researchers, physicians, software engineers, engineers, product developers), it is important to determine the relationship between psychosocial factors and specific organizational outcomes to assist management to determine where to intervene in order to reduce stress and improve employee well-being. Thus, to build confidence in our findings, stress management initiatives should be investigated in other organizational contexts to determine whether the types of intervention strategies implemented by university management are widespread in other work settings. 

In conclusion, the JD-R theoretical framework appears to be evident in the employee-organization relationship in university settings as these findings suggest that universities and other organizations should implement targeted and multilevel resources, such as HR/OHS stress intervention policies and practices that target both the organization and individuals’ needs. As universities have become increasingly large and diverse institutions [28], it is timely to investigate new and emerging psychosocial risks within universities as more evaluative research on work stress interventions is needed at the institutional and sector level to assist management (in universities and other complex organizations) to address issues relating to psychosocial risks and refine or implement new strategies to attain healthy work environments.

## Figures and Tables

**Table 1:** Number and Type of Strategies Implemented and their Target Level.

	University										Total	
Intervention Focus &	A		B		C		D		E		(Rank Order)	
Target Level	N	%	N	%	N	%	N	%	N	%	N	%
Training/Career/Promotion	4	9.8	8	18.2	4	10.8	10	19.6	9	18.8	35	15.8
Organization												
Remuneration/Recognition	5	12.2	9	20.5	11	29.7	4	7.8	6	12.5	35	15.8
Organization												
Work-Life Balance	8	19.5	3	6.8	7	18.9	7	13.7	4	8.3	29	13.1
Organization												
Procedural Justice	4	9.8	5	11.4	5	13.5	7	13.7	6	12.5	27	12.2
Organization												
Job Design/Pressure	5	12.2	3	6.8	0	0	8	15.7	7	14.6	23	10.4
Organization												
Communication/Consultation	5	12.2	5	11.4	0	0	5	9.8	5	10.4	20	9
Organization												
Stress Awareness/Management	3	7.3	3	6.8	5	13.5	4	7.8	3	6.3	18	8.1
Individual & Organization												
Trust in Senior Management	3	7.3	4	9.1	0	0	3	5.9	3	6.3	13	5.9
Organization												
Interpersonal Relations	2	4.9	1	2.3	5	13.5	2	3.9	3	6.3	13	5.9
Individual/Organization Interface												
Job Security/Tenure	1	2.4	2	4.5	0	0	1	2.0	2	4.2	6	2.7
Organization												
Health & Lifestyle	1	2.4	1	2.3	0	0	0	0	0	0	2	0.9
Individual												
TOTAL	41	100	44	100	37	100	51	100	48	100	221	100

**Table 2:** Priority Strategies: Aims, Coverage, Targets, Planning and Implementation Process.

University				
A	B	C	D	E
**Strategy**				
Improved leadership & management skills	Streamlined academic portfolios & integrated support services	Early intervention & management of stress claims	Leadership programs for academics managing departments (coaching, self-awareness, impact of behavior)	Management & leadership development
**Aim**				
Leadership training for supervisors & managers to effectively manage staff	Streamlining administrative systems & processes to eliminate duplication	Increasing awareness of resources for identifying, reporting & managing stress	Improving staff commitment & morale	Improving outcomes (e.g., stress management) by developing leadership skills
**Coverage**				
Supervisors & managers	University-wide	University-wide	Heads of Academic Departments & future Heads of Departments	University-wide
**Target**				
Supervisors & managers	–	All staff including managers	Academic staff only	Managers & Heads of Schools
**Identifying At-risk Groups**				
Working Life survey; feedback from staff & management	Whole of university project	Holistic approach	Previous attempts to introduce program	Focus group findings
**Planning**				
Management & staff	Management & staff	Management & staff	Management only	Management & staff
**Implementation**				
Management & staff	Management & staff	Management & staff	Management, Academic Heads of Department, HR	Management only

**Table 3:** Interventions to Manage Stress, Enhance Job Design, and Address Tenure Concerns.

University				
A	B	C	D	E
**Awareness Raising & Management**				
Updated & promoted EAP Conducted climate/stress surveys	Updated & promoted EAP Updated CISM/counselling program	Updated & promoted EAP Updated stress awareness training (managers) Updated training in stress management (all staff) Updated CISM/counselling program	Updated stress awareness training (managers) Updated training in stress management (all staff) Updated CISM/counselling program Updated training in CISM services (managers)	Updated & promoted EAP Conducted climate/stress surveys
**Job Design**				
Streamlined administration Updated job redesign & classification policies Reviewed job descriptions regularly Updated induction & orientation programs Improved physical work environments	Streamlined administration Updated job redesign & classification policies Updated induction & orientation programs	-	Increased access to resources (academics) Increased staffing (academic & non-academic) Reduced student/staff ratio Streamlined administration Reviewed job descriptions regularly Updated induction & orientation programs Improved physical work environments	Increased access to resources (academics) Reduced student/staff ratio Streamlined student assessment procedures Prioritized job responsibilities Reviewed job descriptions regularly Improved physical work environments Updated performance & development process
**Tenure**				
Improved communication processes for contract renewal	Improved communication processes for contract renewal Updated redundancy & redeployment policies	-	Improved communication processes for contract renewal	Improved communication processes for contract renewal Tightened fixed term contract criteria

Note: EAP = Employee Assistance Program; CISM = Critical Incident Stress Management.

**Table 4:** Interventions to Enhance Work-Life Balance and Communication.

University				
A	B	C	D	E
**Work-Life Balance**				
Established/promoted family-friendly provisions Established/promoted fractional employment & flexible working hours Established/promoted job sharing, 48/52 arrangements, & work from home agreements Monitored absenteeism Encouraged taking accrued leave	Established/promoted family-friendly provisions Established/promoted 48/52 arrangements Monitored absenteeism	Established/promoted fractional employment & flexible working hours Established/promoted rostered days off Established/promoted job sharing, 48/52 arrangements, & work from home agreements Encouraged taking accrued leave	Established/promoted fractional employment & flexible working hours Established/promoted rostered days off Established/promoted job sharing, 48/52 arrangements, & work from home agreements Encouraged taking accrued leave	Established/promoted fractional employment & flexible working hours Established/promoted 48/52 arrangements Encouraged taking accrued leave
**Communication & Consultation**				
Improved management-staff communication Regular updates about organizational objectives More consultation on workplace relations Feedback for good performance Introduced Performance Development Review for all staff	Improved management-staff communication Regular updates about organizational objectives More staff consultation & voice in promotion, performance & redundancy procedures More consultation on workplace relations & organizational change	–	Updated management training in effective communication skills/styles Regular updates about organizational objectives More consultation on workplace relations & organizational change Feedback for good performance	Regular updates about organizational objectives More staff consultation & voice in promotion, performance & redundancy procedures More consultation on workplace relations & organizational change Feedback for good performance

**Table 5:** Interventions to Enhance Workplace Relationships and Trust in Senior Management.

University				
A	B	C	D	E
**Workplace Relations**				
Updated workplace bullying & violence, & discrimination & harassment policies	Updated staff social activities	Updated equal employment opportunity, workplace bullying & violence, & discrimination & harassment policies Updated conflict identification, & mediation & conflict resolution policies	Updated equal employment opportunity, & discrimination & harassment policies	Updated equal employment opportunity, workplace bullying & violence, & discrimination & harassment policies
Trust				
Developed leadership capabilities Training & mentoring of new leaders Increased transparency of management decision making	Developed leadership capabilities Reviewed selection of staff for leadership positions Training & mentoring of new leaders Updated management reporting timeframes	–	Developed leadership capabilities Training & mentoring of new leaders Updated management reporting timeframes	Developed leadership capabilities Reviewed selection of staff for leadership positions Training & mentoring of new leaders

**Table 6:** Interventions to Enhance Training and Promotional Opportunities.

University				
A	B	C	D	E
Training & Promotion				
Updated training in performance management (managers) Updated training for middle managers (management skills) Established peer support systems Mentoring/coaching programs	Updated training in performance, change & time management & interpersonal skills (managers) Updated training for middle managers (management skills) More staff training & development Mentoring/coaching programs Reviewed financial support for higher degree study	More staff training & development Reviewed financial support for higher degree study Reviewed assisted leave for higher degree study Reviewed academics‘ promotion paths	Updated training in performance, change & time management & interpersonal skills (managers) Updated training for middle managers (management skills) More staff training & development Established peer support systems Mentoring/coaching programs Increased career opportunities Reviewed academics‘ promotion paths	Updated training in performance, change & time management & interpersonal skills (managers) Updated training for middle managers (management skills) More staff training & development Mentoring/coaching programs Reviewed financial support for higher degree study Reviewed academics‘ promotion paths

**Table 7:** Interventions to Enhance Fair Procedures, Remuneration, Recognition and Health.

University				
A	B	C	D	E
**Policies & Procedures**				
Reviewed promotion procedures (academics) Updated grievance procedures Updated PMS Increased transparency of performance appraisals	Reviewed recruitment, selection & appointment policies Reviewed promotion procedures (academics) Provided clear promotion criteria & constructive feedback to unsuccessful candidates Increased transparency & fairness of redundancy procedures	Reviewed promotion procedures (academics) Updated grievance procedures Updated PMS Increased transparency of performance appraisals	Reviewed promotion procedures (academics) Updated grievance procedures Updated PMS Increased transparency of performance appraisals	Reviewed promotion procedures (academics) Updated grievance procedures Updated PMS Increased transparency of performance appraisals
**Remuneration & Recognition**				
Updated performance planning & review systems Updated staff reward schemes Developed/improved processes for recognizing teaching excellence Teaching awards Professional development fund	Updated performance planning & review systems Developed/improved processes for recognizing teaching & research excellence VC/teaching & research/long service awards Excellence awards: non-academics Professional development fund	Initiated pay equity measures Updated performance planning & review systems Developed/improved processes for recognizing teaching, research, administration excellence VC/teaching & research/long service awards Excellence awards: non-academics Professional development fund	Updated performance planning & review systems Developed/improved processes for recognizing excellence in teaching Teaching/long service awards	Updated performance planning & review systems Developed/improved processes for recognizing excellence in teaching, administration Teaching & research awards Excellence awards: non-academics
**Health & Lifestyle**				
Established/reviewed meditation program	Established/reviewed fitness & exercise program	–	–	–

Note: PMS = Performance Management System; VC = Vice-Chancellor.

## References

[1] Deloitte Access Economics The Importance of Universities to Australia’s Prosperity; Universities Australia, 2015. https://www.universitiesaustralia.edu.au/ArticleDocuments/783/The%20importance%20of%20universities%20to%20Australia%20s%20prosperity.PDF.aspx.

[2] Health Safety Executive (HSE) Managing the Causes of Work-Related Stress: A Step-by-Step Approach Using the Management Standards. https://safetyresourcesblog.files.wordpress.com/2014/10/managing-the-causes-of-work-related-stress-a-step-by-step-approach-using-management-standards.pdf.

[3] Skakon J., Nielsen K., Borg V., Guzman J. (2010). Are leaders’ well-being, behaviours and style associated with the affective wellbeing of their employees? A systematic review of three decades of research. Work Stress.

[4] Leiter M.P., Bakker A.B., Maslach C. (2014). Burnout at Work: A Psychological Perspective.

[5] Ivancevich J.M., Matteson M.T., Freedman S.M., Phillips J.S. (1990). Worksite stress management interventions. Am. Psychol..

[6] Biron C., Karanika-Murray M. (2014). Process evaluation for organizational stress and well-being interventions: Implications for theory, method, and practice. Int. J. Stress Manag..

[7] Nielsen K., Taris T.W., Cox T. (2010). The future of organizational interventions: Addressing the challenges of today's organizations. Work Stress.

[8] Lamontagne A.D., Noblet A., Spector P., Biron C., Karanika-Murray M., Cooper C. (2012). Intervention development and implementation: Understanding and addressing barriers to organizational-level interventions. Improving Organizational Interventions for Stress and Well-Being: Addressing Process and Context.

[9] Semmer N.K. (2006). Job stress interventions and the organization of work. Scand. J. Work Environ. Health.

[10] Karanika-Murray M., Biron C., Saksvik P.O. (2016). Organizational Health Interventions: Advances in Evaluation Methodology. Stress Health.

[11] Cox T., Taris T.W., Nielsen K. (2010). Organizational interventions: Issues and challenges. Work Stress.

[12] Briner R.B., Reynolds S. (1999). The costs, benefits, and limitations of organizational level stress interventions. J. Organ. Behav..

[13] Nytrø K., Saksvik P.Ø., Mikkelsen A., Bohle P., Quinlan M. (2000). The role and effects of process in occupational stress interventions. Work Stress.

[14] Giga S.I., Cooper C.L., Faragher B. (2003). The development of a framework for a comprehensive approach to stress management interventions at work. Int. J. Stress Manag..

[15] Beehr T.A., Newman J.E. (1998). Research on occupational stress: An unfinished enterprise. Pers. Psychol..

[16] LaMontagne A.D., Keegel T., Louie A.M., Ostry A., Landsbergis P.A. (2007). A systematic review of the job-stress intervention evaluation literature, 1990–2005. Int. J. Occup. Environ. Health.

[17] Giga S.I., Noblet A.J., Faragher B., Cooper C.L. (2003). The UK perspective: A review of research on organizational stress management interventions. Aust. Psychol..

[18] Geurts S., Gründemann R., Kompier M., Cooper C. (1999). Workplace stress and stress prevention in Europe. Preventing Stress, Improving Productivity: European Case Studies in the Workplace.

[19] Burke R.J. (1993). Organizational-level interventions to reduce occupational stressors. Work Stress.

[20] Richardson K.M., Rothstein H.R. (2008). Effects of occupational stress management intervention programs: A meta-analysis. J. Occup. Health Psychol..

[21] Tetrick L.E., Winslow C.J. (2015). Workplace stress management interventions and health promotion. Annu. Rev. Organ. Psychol. Organ. Behav..

[22] DeFrank R.S., Cooper C.L. (1987). Worksite stress management interventions: Their effectiveness and conceptualization. J. Manag. Psychol..

[23] Schaufeli W.B., Taris T.W., Bauer G., Hämmig O. (2014). A critical review of the job demands-resources model: Implications for improving work and health. Bridging Occupational, Organizational and Public Health: A Transdisciplinary Approach.

[24] Bakker A.B., Demerouti E. (2007). The Job Demands-Resources model: State of the art. J. Manag. Psychol..

[25] Demerouti E., Bakker A.B., Nachreiner F., Schaufeli W.B. (2001). The Job Demands-Resources model of burnout. J. Appl. Psychol..

[26] Schaufeli W.B., Bakker A.B. (2004). Job demands, job resources, and their relationship with burnout and engagement: A multi-sample study. J. Organ. Behav..

[27] Dollard M.F., Bakker A.B. (2010). Psychosocial safety climate as a precursor to conducive work environments, psychological health problems, and employee engagement. J. Occup. Organ. Psychol..

[28] Coates H., Goedegebuure L. (2010). The Real Academic Revolution: Why We Need to Reconceptualise Australia's Future Academic Workforce, and Eight Possible Strategies for How to Go About This.

[29] Biron C., Brun J.P., Ivers H. (2008). Extent and sources of occupational stress in university staff. Work J. Prev. Assess. Rehabil..

[30] Catano V., Francis L., Haines T., Kirpalani H., Shannon H., Stringer B., Lozanzki L. (2010). Occupational Stress in Canadian Universities: A National Survey. Int. J. Stress Manag..

[31] Coetzee S.E., Rothmann S. (2005). Occupational stress, organizational commitment and ill health of employees at a higher education institution in South Africa. S. Afr. J. Ind. Psychol..

[32] Kinman G., Wray S. (2014). Taking Its Toll: Rising Stress Levels in Further Education.

[33] Liu C., Spector P.E., Shi L. (2008). Use of both qualitative and quantitative approaches to study job stress in different gender and occupational groups. J. Occup. Health Psychol..

[34] Taris T.W., Schreurs P.J.G., van Iersel-van Silfhout I.J. (2001). Job stress, job strain, and psychological withdrawal among Dutch university staff: Towards a dual-process model for the effects of occupational stress. Work Stress.

[35] Winefield A.H., Jarrett R.J. (2001). Occupational stress in university staff. Int. J. Stress Manag..

[36] Winefield A.H., Boyd C.M., Saebel J., Pignata S. (2008). Job Stress in University Staff: An Australian Research Study.

[37] Gillespie N.A., Walsh M., Winefield A.H., Dua J., Stough C. (2001). Occupational stress in universities: Staff perceptions of the causes, consequences and moderators of stress. Work Stress.

[38] Australian Technology Network (ATN). https://www.atn.edu.au/.

[39] Pignata S., Winefield A.H., Katsikitis M. (2006). Awareness of stress-reduction interventions on organizational attitudes in staff at an Australian university. Proceedings of the 2006 Joint Conference of the Australian Psychological Society and New Zealand Psychological Society.

[40] Eisenhardt K.M. (1989). Building theories from case study research. Acad. Manag. Rev..

[41] Yin R. (1989). Case Study Research: Design and Methods.

[42] Siegrist J. (1996). Adverse health effects of high-effort/low-reward conditions. J. Occup. Health Psychol..

[43] Dollard M.F., Tuckey M.R., Dormann C. (2012). Psychosocial safety climate moderates the demand-resource interaction in predicting work stress. Accid. Anal. Prev..

[44] Morgeson F.P., Dierdorff E.C., Hmurovic J.L. (2010). Work design in situ: Understanding the role of occupational and organizational context. J. Organ. Behav..

[45] Kompier M.A.J., Kristensen T.S., Dunham J. (2001). Organisational work stress interventions in a theoretical, methodological and practical context. Stress in the Workplace: Past, Present and Future.

[46] Croon M.A., van Veldhoven M.J.P.M., Peccei R.E., Wood S., Van Veldhoven M.J.P.M., Peccei R. (2015). Researching individual well-being and performance in context: Multilevel mediational analysis for bathtub models. Well-Being and Performance at Work: The Role of Context.

[47] Kompier M., Cooper C.L. (1999). Preventing Stress, Improving Productivity: European Case Studies in the Workplace.

